# Electrocardiographic Manifestation in Thyrotoxic Periodic Paralysis

**DOI:** 10.7759/cureus.21619

**Published:** 2022-01-25

**Authors:** Maria R Iryaningrum, Ignatius Ivan, Fanny Budiman, Erich Tamio

**Affiliations:** 1 Internal Medicine, School of Medicine and Health Sciences, Atma Jaya Catholic University of Indonesia, Jakarta, IDN

**Keywords:** graves' disease, hyperthyroidism, hypokalemic periodic paralysis, hypokalemia, thyrotoxic periodic paralysis

## Abstract

Thyrotoxic periodic paralysis (TPP) is an unusual complication of hyperthyroidism that may cause diagnostic difficulties due to its clinical feature that may be similar to other diseases. However, TPP can be detected early based on the weakness presentation, which generally affects the lower extremity with proximal muscle involvement, and, additionally, the ECG findings presenting hypokalemia characteristics. This case illustrates a young Indonesian male presenting in the emergency department with paralysis and typical ECG findings suggesting TPP. Early identification of TPP is necessary for executing proper treatment and reducing complications.

## Introduction

Thyrotoxic periodic paralysis (TPP) is characterized by short-term recurring bouts of flaccid muscular paralysis affecting the proximal muscles more than the distal muscles [1]. Weakness is almost always accompanied by hypokalemia, and the severity is correlated with the degree of hypokalemia [1,2]. The most common etiology of TPP is due to Graves' disease, but other conditions, such as subacute thyroiditis, toxic nodular goiters, thyroid-stimulating hormone (TSH)-secreting tumor, amiodarone-induced thyrotoxicosis, and factitious hyperthyroidism, can also induce TPP [3-6]. The underlying mechanism in TPP is involving a hyperadrenergic, hyperthyroid state that stimulates sodium-potassium (Na/K) ATPase pump activity leading to intracellular potassium shift and hypokalemia [7]. The triad of ECG manifestation in TPP includes resting sinus tachycardia, prolonged QT-U intervals, and prolonged PR intervals [8]. We report a case of a 31-year-old Indonesian male presenting to the emergency department with acute onset of all extremity weakness.

## Case presentation

A 31-year-old male accompanied by his relative presented to the emergency department in a wheelchair. He complained about his sudden limb weakness on the entire upper and lower extremities. The lower extremities were reported to be more severe compared with the upper extremities. He was on his bed at around 1 AM when he suddenly felt a weakness over his entire extremities. He was unable to move his extremities, thus preventing him from getting up from his bed. During the episode, he complained of palpitation and excessive sweating, yet he was still conscious. The patient denied any previous strenuous exercises, food with a lot of sugars or carbohydrates, licorices, stress, and cold temperatures that may trigger the events.

This sudden attack was also previously felt three weeks ago when he was going to the bathroom in the morning and suddenly felt a similar limb weakness, making him fall to the ground. The attack was also accompanied by palpitation and diaphoresis. During this previous attack, he was still able to get up and walk. Later during the afternoon on the same day, he visited a physician and was prescribed methylprednisolone 16 mg, omeprazole 20 mg, mecobalamin 500 mg, and paracetamol 500 mg for five days. In addition to his complaints, he noticed a slight tremor that has persisted for three weeks, which appears every time he grabs an object. The patient experienced 5 kg weight loss in the last two months.

The patient denies any history of trauma, nausea and vomiting, paresthesia, dizziness, headache, blurred vision, tinnitus, chest discomfort, dysphagia, anosmia, and fever. There were no changes in the frequency and quality of defecation and urination before and after the first episode. The patient denied any drug abusive behavior or alcohol consumption. Similar complaints in the family were denied by the patient.

**Figure 1:**
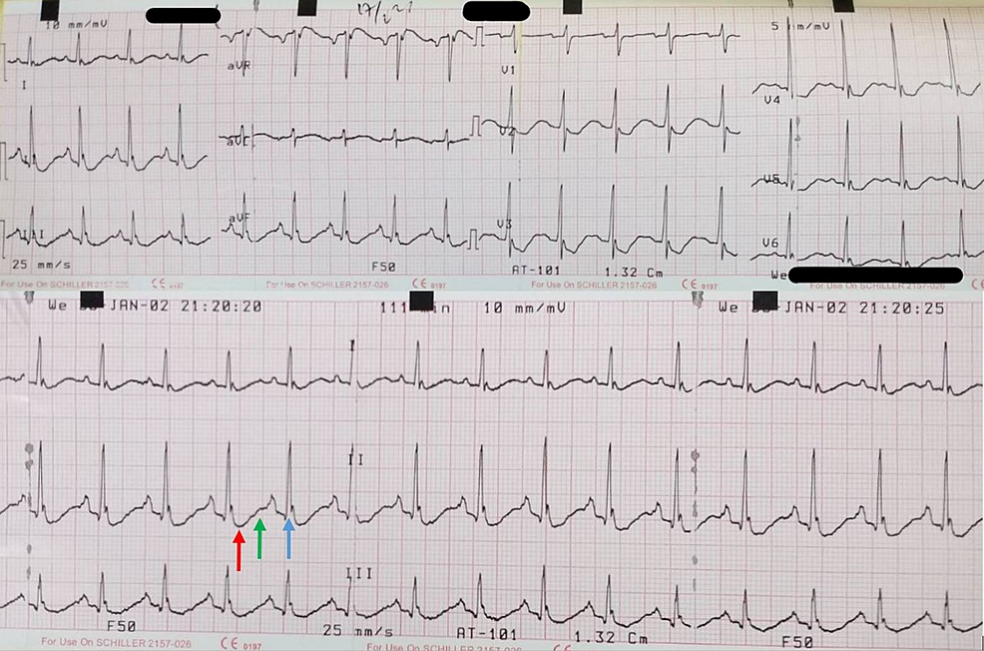
Initial ECG Showing Sinus Tachycardia and Hypokalemia Features Including ST Depression (Red Arrow), U Wave (Green Arrow), and High QRS Voltage (Blue Arrow)

Upon physical examination, he was alert (Glasgow Coma Scale score: 15), with blood pressure of 100/70 mmHg, and he was tachycardic (120 BPM). His body mass index was 21.4, with mid-upper arm circumference of 23. No exophthalmos was identified. An enlarged thyroid gland was observed upon inspection, which measures 6 × 3 cm when palpated and is soft in consistency. No bruit was heard upon auscultation. In addition to the fine tremor, the patient had a reduced motoric strength of 1/5 in all four of his extremities. There were no sensory or cranial nerve deficits. Deep tendon reflexes were missing. Babinski sign and lateralization were negative. The ECG evaluation showed sinus tachycardia and ST depression, U wave, and high QRS voltage, which is consistent with hypokalemia characteristics (Figure 1). A nerve conduction velocity test was not performed. On laboratory examination, his serum potassium level was 1.26 mmol/L. Other laboratory results such as hematological examination, blood gas analysis, random blood glucose, urea, creatinine, and estimated glomerular filtration rate showed a normal result. A diagnosis of TPP secondary to Graves' disease was suspected.

**Figure 2:**
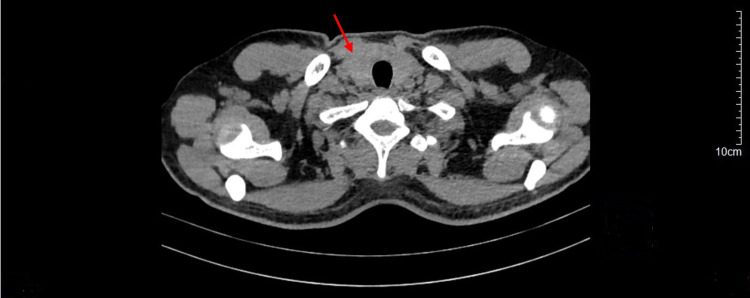
Axial View of Chest Non-Contrast CT Scan Showing Enlarged Right Thyroid Lobe (Red Arrow)

The patient was administered 500 cc of ringer lactate mixed with 25 mEq of KCl every four hours, repeated up to six times. Then, 150 cc of KCl was administered intravenously, and one tablet of KCl 1200 mg was given orally. Additionally, intravenous lansoprazole 30 mg was also administered to prevent gastrointestinal stress ulcer. Propranolol was temporarily not administered considering a nearly borderline low blood pressure. Serum potassium level was obtained the day after, showing an improvement with 4.46 mmol/L. Moreover, the TSH level showed a result of 0.60 µIU/mL and free T4 of 2.59 ng/dL. A further evaluation with non-contrast CT scan showed enlargement of the right thyroid lobe (Figure 2). The patient was then transferred to the inpatient ward after stabilization and was given methimazole 20 mg daily in order to maintain an euthyroid condition. Surgical management was not considered because conservative therapy was in preference. The patient was diagnosed with Graves' disease and discharged on the following day with antithyroid medication.

## Discussion

TPP is more prevalent in Asian populations, with males being more susceptible than women [9]. Despite the fact that women are more usually diagnosed with thyroid illness, the male-to-female ratio of TPP is 4-20:1. TPP may sometimes provide diagnostic difficulties for physicians, since various types of paralysis may be related to hypokalemia (Table 1) [1]. Among other differential diagnoses, the most similar presentation is familial periodic paralysis (FPP) [1]. However, FPP is most often diagnosed in Caucasians throughout their first and second decades of life and is inherited as an autosomal dominant trait, while TPP is most often diagnosed between the third and fifth decades and without a family history of similar presentation [1].

**Table 1:** Differential Diagnosis of Acute Onset of Diffuse Muscle Weakness

Various types of paralysis that may be related to hypokalemia [1]
Familial periodic paralysis
Idiopathic periodic paralysis
Severe thyrotoxic myopathy
Guillain-Barré syndrome
Polymyositis
Myasthenia gravis
Tick paralysis
Acute intermittent porphyria
Infectious myositis (human immunodeficiency virus, anterior poliomyelitis, West Nile virus)
Drug overdose/poisoning (β-agonist drugs, theophylline, organophosphates)
Severe hypokalemia (diuretic abuse, Bartter syndrome, acute gastroenteritis, primary hyperaldosteronism, renal tubular acidosis)

The clinical features of TPP involve weakness of the lower extremities, which are mainly affected compared with upper extremities, and the proximal muscles, which are more afflicted than the distal muscles [9]. Abrupt onset of paralysis commonly occurs as a result of strenuous exercise or consumption of a high-carbohydrate meal [1]. Muscular weakness may vary from minor to complete paralysis and with no concomitant sensory or cognitive dysfunction [1]. Typically, deep tendon reflexes are missing or diminished [1].

ECG changes in TPP are typically associated with hypokalemia, including U waves, QRS widening, QT prolongation, and T wave flattening, in addition to sinus tachycardia, tachyarrhythmias, or in some cases atrioventricular blocks or arrest [10]. Findings such as sinus tachycardia indicating hyperadrenergic state, prolonged QT-U interval indicating hypokalemia, and paradoxically prolonged PR interval in the sinus tachycardia state that might indicate thyrotoxicosis are the triad of typical ECG findings in TPP [8]. Moreover, high QRS voltage has been associated with TPP [11]. In the present case, ECG showed sinus tachycardia and ST depression, U wave, and prolonged QT-U interval with high QRS voltage, suggesting a typical ECG presentation in TPP cases [8,12]. Considering these ECG criteria with the weakness presentation can help in the early detection of TPP prior to obtaining laboratory results and, furthermore, avoiding aggressive measures to replenish potassium levels, which may possibly result in rebound hyperkalemia and deadly conduction abnormalities [8].

Laboratory findings aside from hypokalemia include increased free T4 and free T3 with decreased TSH serum, hypophosphatemia, hypomagnesemia, increased creatine phosphokinase, and normal acid-base balance [1]. Other urinalysis findings include spot urine potassium excretion of less than 20 mmol/L, potassium-to-creatinine ratio of less than 2, transtubular potassium gradient of less than 3, hypercalciuria, hypophosphaturia, and urine calcium-to-phosphorus ratio of more than 1.7 [1].

The first approach in managing TPP is to restore paralysis, and the next step is to avoid future episodes by establishing an euthyroid condition [1]. While treating an acute attack involves hypokalemia correction to prevent catastrophic cardiac arrhythmias, clinicians must realize that patients with TPP do not have a complete body potassium deficit [1]. This is due to the fact that hypokalemia is not the result of potassium losses, but instead, it is because of intracellular potassium shifting [1]. Therefore, aggressive therapy may cause hyperkalemia, and routine potassium supplementation is not beneficial [1,9]. The use of oral or parenteral potassium (~60-120 mEq) is effective in restoring paralysis. A study by Singhai et al. reported that those receiving potassium supplementation regained muscular strength more quickly than those receiving regular saline (6.3 versus 13.5 hours) [13]. It should also be noted that potassium should not be administered in dextrose-containing solutions because it stimulates insulin release, which may exacerbate weakness caused by potassium influx [9]. Additionally, nonselective β-blockers reduce not only thyrotoxic symptoms but also paralysis by reducing potassium's intracellular shift [14]. Propranolol (80-240 mg/day) has been administered prophylactically and has been shown to significantly lower the occurrence of spontaneous TPP episodes [15,16]. Meanwhile, there is no protection for patients from paralytic episodes by selective β-blockers because hampering the β2 receptor is essential in mediating the catecholamine-induced increase in skeletal muscle Na/K ATPase activity [17].

The next important aspect is successful thyrotoxic management based on the underlying cause since paralytic episodes terminate when an euthyroid condition is restored [1]. Until an euthyroid condition is maintained, intense activity, alcohol, and high-carbohydrate meals should be avoided [18]. Thyrotoxicosis induced by high thyroid hormone intake is managed by stopping the related drug [19]. Moreover, in a rare case in which TPP was manifested in pregnancy with hyperemesis gravidarum, the resolution of the hyperemesis would also resolve hyperthyroidism [20]. Treatment with radioiodine ablation or thyroidectomy should be started promptly in cases of Graves' disease, toxic multinodular goiter, or toxic adenoma [1,9]. In addition, methimazole or propylthiouracil should be given while awaiting definitive therapy [1,9].

The mechanism through which hyperthyroidism results in TPP is unknown. Thyroid hormone enhances tissue response to β-adrenergic stimulation, which in turn enhances sodium-potassium ATPase activity on the skeletal muscle membrane [21]. This often results in the entry of potassium into cells, perhaps resulting in hyperpolarization of the muscular membrane and relative inexcitability of the muscle fibers. Patients with TPP were reported to have increased sodium pump activity compared to thyrotoxic patients who did not experience paralytic episodes [22]. Thus, increased thyroid hormone may predispose to paralytic episodes by increasing vulnerability to epinephrine or insulin's hypokalemic effect [23].

## Conclusions

TPP is commonly present in young Asian males and likely to be missed initially. In spite of the importance of obtaining thyroid function tests immediately, early diagnosis based on clinical manifestations and ECG examination might help clinicians plan patient therapy timely and thereby improve prognosis.
